# The Book World of Medicine and Science

**Published:** 1899-08-26

**Authors:** 


### The Book World of Medicine and Science.

Intestinal Obstruction: Its Varieties, with their Pathology, Diagnosis, and Treatment. By Frederick Treves, F.R.C.S. With 118 Illustrations. New and Revised Edition. (London: Cassell and Co. 1899. Price 21s.)

This is said to be a new edition, but practically it is a new book, and one the contents of which from beginning to end should be thoroughly mastered by everyone who aims at doing good work in his profession. Naturally the treatment of such a mechanical difficulty as obstruction to the onflow of the intestinal contents must to a large extent be itself mechanical—in other words, operative—and, therefore, if it is to be successfully carried out, must fall into the hands of surgeons who are prepared to undertake operative procedures; but diseases of the abdominal contents form so large a proportion of the cases which come before every practitioner, and obstruction is so dangerous a complication or termination of these diseases, that no one can afford to remain ignorant of the lessons so well taught in such a book as this, being, as they evidently are, the outcome of a large amount of study of the subject and a wide practical experience. Referring to the causes of obstruction, Mr. Treves says that the records of the London Hospital show that the cases ascribed to fæcal accumulation are the most numerous; then come, in order, cases of stricture of large intestine, intussusception, strangulation by bands, obstruction due to tumours external to the bowels, blocking of of the gut by gall-stones or foreign bodies, and then other forms of obstruction, all of which may be spoken of as rare. We were hardly prepared to find fæcal accumulation stand so high in the list. Interesting as the whole of this book is, as presenting a complete survey of the pathology and symptomatology of intestinal obstruction, it is to the part devoted to treatment that the majority of its readers will probably most frequently turn for help in their daily work. And they will not be disappointed. The lines to be followed are carefully laid down, and, although the difficulty of diagnosis as to the exact conditions existing in any given case has always to be reckoned with, great assistance is given by the manner in which the author narrows down the problem to a consideration of the really very few measures which are capable of exercising any effective influence upon the result. The measures which have to be considered under the head of treatment may be dealt with under the following heads: (1) Rest; (2) the administration of morphia; (3) the evacuations of the lower bowel; (4) the question of feeding; (5) the use of measures other than operations; and (6) the treatment by operation. On all these the directions given are admirably clear. Morphia, it is said, is an absolute necessity in acute intestinal obstruction, and should be administered with as little delay as possible, for it "eases the agonising pain and gives the patient a respite from what is, without doubt, one of the most terrible forms of human suffering." Moreover, morphia restores for the time being a state of peace within the abdomen, and puts a stop to the disordered peristaltic movements which play so large a part in the production and aggravation of some forms of acute obstruction. Indeed, in a not inconsiderable number of cases strangulation by band has become unlocked under its influence. It is always to be remembered, however, that the administration of morphia may seriously obscure the diagnosis. Its very efficacy in giving relief to the more urgent symptoms constitutes the great danger to be apprehended from its too free, and especially from its repeated administration, for "it may so modify the symptoms and so affect the general aspect of the case that the more characteristic manifestations of the malady are put in entire abeyance." "I have been called," says Mr. Treves, "to see patients who were actually dying, and in whom an autopsy revealed a coil of strangulated and utterly gangrenous bowel. These patients had been dosed from the first with morphia, and had died without giving a sign. The suggestion that they were dying of strangulation of the bowel has been met with the question, 'Where are the symptoms?'." In regard to feeding in cases of acute intestinal obstruction Mr. Treves speaks very positively: "It may be said, in general terms, that the question of feeding does not arise. The patient should be starved, not fed," and he points out that one of the chief causes of death in such cases is the absorption of poisonous material from the loaded bowel. If anything is to be given by the mouth it should be a little *hot* water or a little *hot* weak tea. The sucking of ice is to be condemned without reservation." Measures other than operation call, says Mr. Treves, for very little comment. "They are for the most part feeble excuses for avoiding or delaying an operation." We then come to the important question of operation. There is no doubt about the extreme responsibility attaching to those who have to advise in these anxious cases. "There is no avoiding the fact that acute intestinal obstruction, if unrelieved, ends in death." It is perfectly true that there are isolated examples of spontaneous recovery, "but when the whole mass of cases is considered the number of those examples of recovery is so miserably few that they form no kind of ground for depriving the patient of a hope of life by operation." Even in intussusception "there can be no shadow of a doubt that the risk of operation" is infinitely less than that of leaving the case alone. Laparotomy then should be done "at the earliest possible moment—as soon, indeed, as the diagnosis is reasonably clear," and it is added that "in cases of acute abdominal trouble in which the diagnosis is not clear the better and safer course is to operate." Now for some points in regard to the operation. Mr. Treves advises that where it is possible the stomach should be washed out before the patient is placed on the table. In bad cases, which are pretty well under morphia, very little anæsthetic may be necessary; perhaps nothing beyond cocaine or eucaine. A medium incision should be employed. "Experience is decidedly against the making of the incision over the supposed site of the obstruction." In extreme cases, if by the insertion of two fingers the cause cannot be discovered, a simple enterostomy may be performed. The interference is very slight, and may be performed under pressing circumstances without an anæsthetic. But, except in extreme cases, Mr. Treves has no love for this "very rough and ready operation," against which "a vast array of arguments may be advanced." Indeed, he says that as surgery advances "this extreme, irrational, and blindly devised operation will become less and less frequent." In searching for the obstruction he warns his readers against a small incision. "The incision must be as large as may be necessary to deal with the condition without unnecessary handling of the distended coils," and if it becomes necessary to allow the bowels to protrude "there must be no stint" in enlarging the incision. Full directions regarding many details in the operation are given, and it is especially insisted on that *in advanced cases* the operation must not be looked upon as complete until the distended gut has been evacuated, for in such cases the immediate danger to life "lies rather with the poisonous material in the intestine than with the actual obstructing cause beyond its walls."

But we must stop. The book is the work of a practical surgeon, whose lead may safely be followed in the very trying class of cases of which it treats. It certainly is worthy of the most careful study.

